# Fabrication of an Efficient N, S Co-Doped WO_3_ Operated in Wide-Range of Visible-Light for Photoelectrochemical Water Oxidation

**DOI:** 10.3390/nano12122079

**Published:** 2022-06-16

**Authors:** Dong Li, Fachao Wu, Caiyun Gao, Hongfang Shen, Fei Han, Fenglan Han, Zhanlin Chen

**Affiliations:** 1 School of Material Science and Engineering, North Minzu University, Yinchuan 750021, China; shen_hongfang@nun.edu.cn (H.S.); hanfei@nun.edu.cn (F.H.); 2002074@nun.edu.cn (F.H.); 18995091369@163.com (Z.C.); 2 Chemical Science and Engineering College, North Minzu University, Yinchuan 750021, China; wufachao1997@163.com; 3 International Scientific & Technological Cooperation Base of Industrial Waste Recycling and Advanced Materials, Yinchuan 750021, China

**Keywords:** N, S co-doped, water oxidation, tungsten trioxide, photoanode, photoelectrochemical, water splitting

## Abstract

In this work, a highly efficient wide-visible-light-driven photoanode, namely, nitrogen and sulfur co-doped tungsten trioxide (S-N-WO_3_), was synthesized using tungstic acid (H_2_WO_4_) as W source and ammonium sulfide ((NH_4_)_2_S), which functioned simultaneously as a sulfur source and as a nitrogen source for the co-doping of nitrogen and sulfur. The EDS and XPS results indicated that the controllable formation of either N-doped WO_3_ (N-WO_3_) or S-N-WO_3_ by changing the n_W_:n_(NH4)2S_ ratio below or above 1:5. Both N and S contents increased when increasing the n_W_:n_(NH4)2S_ ratio from 1:0 to 1:15 and thereafter decreased up to 1:25. The UV-visible diffuse reflectance spectra (DRS) of S-N-WO_3_ exhibited a significant redshift of the absorption edge with new shoulders appearing at 470–650 nm, which became more intense as the n_W_:n_(NH4)2S_ ratio increased from 1:5 and then decreased up to 1:25, with the maximum at 1:15. The values of n_W_:n_(NH4)2S_ ratio dependence is consistent with the cases of the S and N contents. This suggests that S and N co-doped into the WO_3_ lattice are responsible for the considerable redshift in the absorption edge, with a new shoulder appearing at 470–650 nm owing to the intrabandgap formation above the valence band (VB) edge and a dopant energy level below the conduction band (CB) of WO_3_. Therefore, benefiting from the S and N co-doping, the S-N-WO_3_ photoanode generated a photoanodic current under visible light irradiation below 580 nm due to the photoelectrochemical (PEC) water oxidation, compared with pure WO_3_ doing so below 470 nm.

## 1. Introduction

The development and utilization of hydrogen energy is considered to be one of the significant ways to resolve the energy crisis and environmental pollution [1,2,3].

At present, there are many strategies to produce hydrogen by solar energy, including electrolytic and solar thermal water splitting, PEC water splitting, and so on [4]. Among them, PEC water splitting could directly convert abundant solar energy into clean hydrogen energy. Therefore, it is regarded as one of promising ways and has attracted considerable attention since the TiO_2_ photoanode was first reported by Honda and Fujishima [5,6,7,8,9,10]. However, the half-reaction of PEC water oxidation on photoanode is considered to be a key process to affect the efficiency of fuel generation due to the difficult kinetic nature. Moreover, the bandgap of TiO_2_ is too wide (3.0–3.2 eV) to respond to the visible light of sun spectrum, being consequently responsible for low efficiency in the utilization of solar light. So, it is of great importance to develop a stable and robust semiconductor photoanode with narrow bandgaps to enhance the absorption of solar light.

So far, intensive research has focused on the development of efficient semiconductor photoanodes, such as WO_3_ [8,11,12,13,14,15], α-Fe_2_O_3_ [9,16,17,18,19], ZnO [20,21], Cu_2_O [22,23], and Ta_3_N_5_ [24,25,26] for PEC water oxidation.

Since WO_3_ was reported as a PEC photoanode by Hodes in 1976 [27], it has attracted immense attention because of its visible light response (bandgap, E_g_ = 2.6–2.8 eV), strong absorption within the solar spectrum and good photochemical stability under acidic conditions. However, as the WO_3_ photoanode cannot respond to visible light above 460 nm, its solar spectrum utilization is still low. Taking this disadvantage into account, enhancing the light absorption at longer wavelengths is the key to improving the solar energy conversion efficiency of the WO_3_ photoanode. Therefore, extension of light absorption to longer wavelengths by bandgap engineering of WO_3_ is an important and interesting research subject in the related field. 

Doping WO_3_ with transition metals (Ti, Fe, Co, Ni, Cu, Zn) [28,29] and other metals (Mo, Dy, Te, Ta, V, Yb, Ce) [30,31,32,33,34,35,36] was reported to improve not only the light absorption at longer wavelengths but also the PEC performance. Unfortunately, the PEC performance of WO_3_ photoanodes doping with metallic dopants decreases with increasing doping concentration and can be even lower than pure WO_3_ owning to recombination center generation.

In recent years, the research mostly focused on single doping WO_3_ with selective nonmetallic elements (C, N, S) [37,38,39,40], as well as molecules (N_2_, Xe and CO) [41,42,43] to enhance the light absorption. However, attention has scarcely been focused on the multielement co-doped WO_3_ yet so far. We noted that co-doped with two or more nonmetallic elements was widely reported in TiO_2_ systems [44,45,46,47,48,49,50], where the photocatalytic activities of TiO_2_ were further improved compared to single doping due to their excellent visible light photocatalysis caused by the narrowed bandgap. This indicated that nonmetallic element co-doped TiO_2_ could enhance the visible light, but also reduce the recombination rate of photo-induced electron-hole pairs. WO_3_ exhibits property similar to that of TiO_2_ because the VB of WO_3_ and TiO_2_ are mainly composed of O 2p orbitals. It is confirmed that the effective nonmetallic doping induces hybridization of the outer orbitals of the doped elements and the VB of TiO_2_ to form a new energy level at the top of the VB and reduce the bandgap of TiO_2_. This suggests that co-doping of WO_3_ with two or more nonmetallic elements is a promising route to improve the absorption efficiency of WO_3_. 

Herein, we reported the first simultaneous synthesis of S-N-WO_3_ using (NH_4_)_2_S as N and S atom source. In this strategy, S-N-WO_3_ exhibited a narrower energy bandgap compared with the pure one. It is attributed to the delocalization of the N 2p orbit with the O 2p orbit after doping of N. Furthermore, S-N-WO_3_ extended its optical response range to longer wavelength visible light because of the fact that 3s (S^6+^) orbitals can be delocalized with W 5d and O 2p orbitals to form a new intermediate level above the VB top. Therefore, the absorption threshold of S-N-WO_3_ can be lowered by co-doping with the S and N elements. Based on this transition, the performance of S-N-WO_3_ for PEC water oxidation is superior to that of pure WO_3_.

## 2. Materials and Methods

### 2.1. Materials

Tungstic acid (H_2_WO_4_), Marpolose (60MP-50), and Polyethylene glycol (PEG, molecular weight = 2000) were purchased from Aladdin’s Reagent (Shanghai Aladdin Bio-Chem Technology Co., Ltd, Shanghai, China) and (NH_4_)_2_S was purchased from Macklin Reagent (Shanghai Macklin Biochemical Co.,Ltd., Shanghai, China). A Fluorine-doped tin oxide (FTO)-coated glass substrate was obtained from Dalian HeptaChroma Co., Ltd. (Dalian, China); Millipore water (DIRECT-Q 3UV, Merck Ltd., Shanghai, China) was used for all the experiments. All other chemicals were of analytical grade, and they were used as received, unless mentioned otherwise.

### 2.2. Synthesis of S-N-WO_3_

A total of 1.36 mL (NH_4_)_2_S (20.0 mmol) were drop by drop added to 1.0 g H_2_WO_4_ (4.0 mmol) under vigorous stirring at room temperature to form blue solution with molar ratio (n_W_:n_(NH4)2S_) of H_2_WO_4_ and (NH_4_)_2_S of 1:5–25. After continuous stirring for 15 min, the solvent was slowly evaporated to yield a (NH_4_)_2_S-derived precursor. The (NH_4_)_2_S-derived precursor powders were calcined at 450 °C (1 °C min^−1^) for 1.5 h in flowing O_2_ to obtain different WO_3_ samples, which are denoted as WO_3_–5, WO_3_–10, WO_3_–15, WO_3_–20, and WO_3_–25, respectively. A pure WO_3_ sample denoted as WO_3_–0 was prepared in the same manner without addition of (NH_4_)_2_S.

### 2.3. Fabrication of Electrodes

In a typical procedure, an (NH_4_)_2_S-derived precursor powder (800 mg), PEG (400 mg), and Marpolose (80 mg) were mixed in water (0.6 mL) under slow stirring for 4 h to form a smooth paste without bubbles. The resulting paste was squeezed on a clean FTO glass substrate by a doctor-blade coater and dried at 80 °C for 15 min. After repeating the procedure two times, the electrodes were calcined at 450 °C in O_2_ flow for 1.5 h to give different WO_3_ electrodes. The pure WO_3_ electrode was fabricated by the same method using a precursor prepared without addition of (NH_4_)_2_S.

### 2.4. Measurement

Powder X-ray diffraction (XRD) were measured by a Shimadzu XRD-6000 diffractometer (Shimadzu International Trade (Shanghai) Co., Ltd., Shanghai, China) using monochromated Cu Kα (*λ* = 1.54 Å) radiation. The energy-dispersive X-ray spectroscopic (EDS) data were taken using an electron probe microanalysis (JED-2300, JEOL, Tokyo, Japan) operated at an accelerating voltage of 10 kV. Raman spectra were taken using a Raman microspectroscopic apparatus (Horiba-Jobin-Yvon LabRAM HR, Paris, France). The XPS spectra were recorded using a Thermo Fisher Scientific ESCALAB Xi+ instrument (Thermo Fisher Scientific (China) Co., Ltd., Shanghai, China) and calibrated in reference to C 1 s peak fixed at 284.2 eV. UV-visible diffuse reflectance spectra (DRS) were recorded on a spectrophotometer (Shimadzu UV-2700, Shimadzu International Trade (Shanghai) Co., Ltd., Shanghai, China).

All PEC measurements were examined in a two-compartment PEC cell separated by a Nafion membrane using an electrochemical analyzer (Shanghai Chenhua Instrument Co., Ltd., Shanghai, China, CHI660E). A three-electrode system was employed using different WO_3_ electrodes and Ag/AgCl electrodes in one cell as the working and reference electrodes, respectively, and a Pt wire—in the other cell as the counter electrode. The linear sweep voltammograms (LSV) were measured at a scan rate of 5 mV s^−1^. Light (λ > 450 nm, 100 mW cm^−2^) was irradiated from the backside of the working electrode using a 500 W xenon lamp with a UV-cut filter (λ > 450 nm). The output of light intensity was calibrated as 100 mW cm^−2^ using a spectroradiometer (USR-40, Ushio Shanghai Inc., Shanghai, China). Photoelectrocatalysis was conducted under potentiostatic conditions at 0.5 V at 25 °C with illumination of light (λ > 450 nm, 100 mW cm^−2^) for 1 h. All the PEC experiments were carried out under argon atmosphere in an aqueous 0.1 M phosphate buffer solution (pH 6.0). The amounts of H_2_ and O_2_ evolved were determined from the analysis of the gas phase of counter and working electrode compartments, respectively, using gas chromatography (Shimadzu GC-8A with a TCD detector and molecular sieve 5 A column and Ar carrier gas). A monochromic light with 10 nm bandwidth was provided by a 500 W xenon lamp using a monochromator for incident photon-to-current conversion efficiency (IPCE) measurements.

## 3. Results

### 3.1. Characterization Structure of S-N-WO_3_

**Figure 1:**
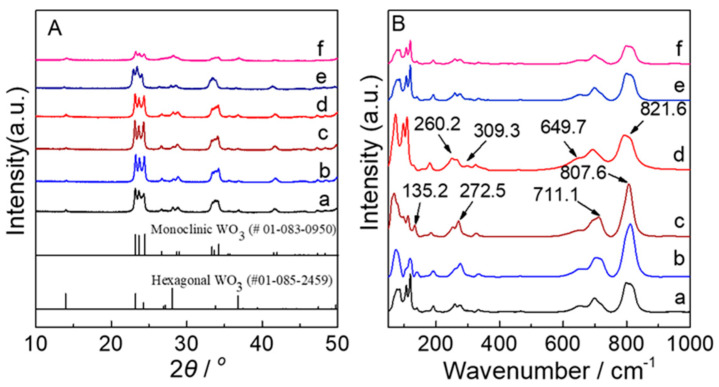
(**A**) XRD patterns and (**B**) Raman spectra of (a) WO_3_−0, (b) WO_3_−5, (c) WO_3_−10, (d) WO_3_−15, (e) WO_3_−20, and (f) WO_3_−25.

The phase composition of the WO_3_ samples calcined at 450 °C were ascertained by XRD (Figure 1A) and Raman (Figure 1B) measurements. In Figure 1A, it can be clearly observed that all of the samples exhibited the relatively weak peaks at 14.0°, 28.1°, and 36.8° corresponding to (100), (200), and (202) planes, respectively, which can be assigned to a hexagonal WO_3_ crystalline phase (PDF # 01-085-2459) [41]. Alongside the hexagonal peaks, the main peaks at 23.1°, 23.7°, 24.3°, 26.6°, 28.7°, 29.1°, 33.3°, 33.8°, and 41.3° for a monoclinic WO_3_ crystalline phase (PDF # 01-083-0950) [15], consisting of the (002), (020), (200), (120), (112), (022), (202), (220), and (222) plane, respectively. Especially, it can be seen that the crystallinity of S-N-WO_3_ samples decreases with an increasing n_W_:n_(NH4)2S_ ratio over 1:15, suggesting that the crystalline structure of S-N-WO_3_ samples can be strongly affected by the addition of (NH_4_)_2_S. 

Raman spectra of the WO_3_ samples exhibited the characteristic peaks of the monoclinic WO_3_ at 135.2 cm^−1^ (lattice vibration), 272.5 cm^−1^ (δ (O-W-O) deformation vibration), 711.1 cm^−1^ and 807.6 cm^−1^ (ν (O-W-O) stretching vibration) in the 100~1000 cm^−1^. Meanwhile, the characteristic peaks for the hexagonal WO_3_ at 260.2 cm^−1^ and 309.3 cm^−1^ (δ (O-W-O) deformation vibration), 649.7 cm^−1^ and 821.6 cm^−1^ (ν (O-W-O) stretching vibration) were observed. The Raman analysis also shows the tendency of Raman peaks broadening due to overdoping from the n_W_:n_(NH4)2S_ ratio of 1:15, which is in agreement with the XRD results.

**Figure 2:**
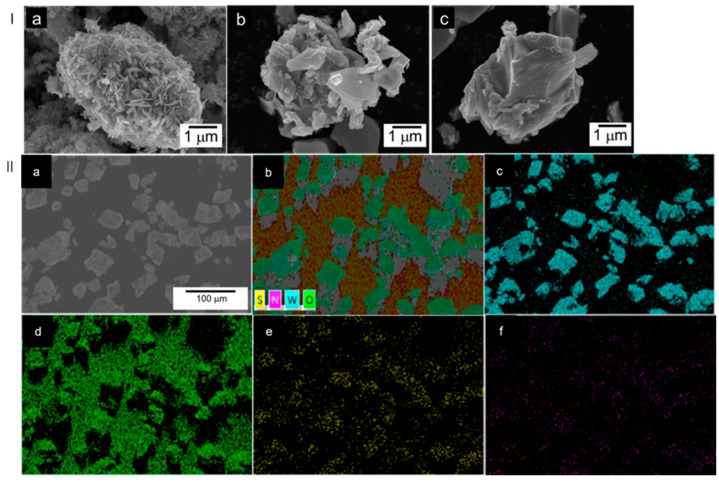
(**I**). SEM images of (**a**) WO_3_−0, (**b**) WO_3_−5, and (**c**) WO_3_−15 samples, respectively; (**II**) (**a**) SEM_EDX elements distribution mapping images of the WO_3_−15; (**b**) W, O, S, N layered, (**c**) W, (**d**) O, (**e**) S, and (**f**) N, respectively.

As shown in Figure 2I, it can be clearly observed that the morphologies of WO_3_−5 and WO_3_−15 samples (Figure 2Ib,c) are different from that of WO_3_−0 (Figure 2Ia) composed of nanosheet of ca. 5 μm. It also should be noted that the particles showed the trend of agglomeration with increasing addition of (NH_4_)_2_S. For the WO_3_−5 sample (Figure 2Ib), it mainly consisted of microparticles of about 0.7–1.8 μm, while the WO_3_−15 sample (Figure 2Ic) was uniformly made up of blocklike particles of about 5.2 μm in size. EDX analyses were taken to confirm the presence of S and N elements. The elemental maps of the EDX for the WO_3_−15 sample are shown in Figure 2II, where the uniform distribution of W and O (Figure 2IIc,d) are confirmed. While the signals of both S and N can be clearly detected on the same structural portion, no other impurity elements were observed in the samples. However, both N and S mappings exhibited higher distribution due to the presence of higher contents in the WO_3_−15 sample. The atom number ratios of W/N as well as W/S were calculated from EDS data to exhibit that it increases with an increase in the n_W_:n_(NH4)2S_ ratio from 1:0 to 1:15 and thereafter decreased above 1:15 (Figure S1 and Table 1).

**Figure 3:**
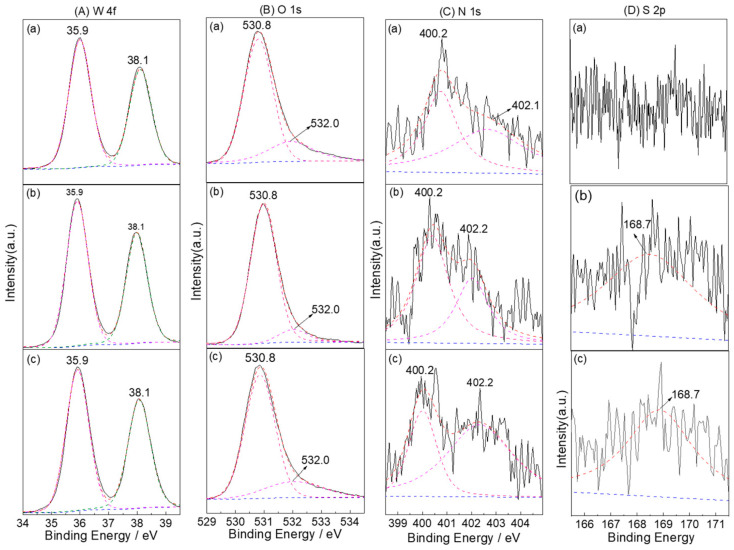
XPS spectra of (**a**) WO_3_−5, (**b**) WO_3_−10, and (**c**) WO_3_−15 in (**A**) W 4f, (**B**) O 2p, (**C**) N 1s, and (**D**) S 2p regions.

The chemical composition and valence states of different WO_3_ samples were investigated through XPS. The spectra were calibrated with the C 1s peak as reference. As shown in Figure S2, the XPS survey spectrum of WO_3_-0 depicts that no other impurity signals, besides the C 1s line, were detected and only W and O. The high-resolution XPS spectrum of W 4f exhibited two peaks at 37.7 eV and 35.5 eV associated with the spin-orbit doublet of W 4f_7/2_ and W 4f_5/2_, respectively, for a W^6+^ state in WO_3_ [11,51]. The apparent peaks at 531.0 eV and 530.2 eV in the XPS spectrum of O 1s can be assigned to the H_2_O and W-O species, respectively [52,53]. The XPS spectra of W 4f doublet for WO_3_−5, WO_3_−10, and WO_3_−15 samples are shown in Figure 3A. Three of the samples exhibited two characteristic peaks at 38.1 eV and 35.9 eV corresponding to 4f_5/2_ and W 4f_7/2_ components of the WO_3_ lattice similar to WO_3_−0. The components with binding energies 530.8 and 532.0 eV in the high-resolution O 1s spectra (Figure 3B) are correspondent to the W-O and hydrocarbonate species, respectively. The XPS spectrum in an N 1S region of 399–404 eV (Figure 3C) exhibited two peaks at 400.2 eV and 402.2 eV, as obtained by two-bands deconvolution. The former one is ascribed to the binding energies of W-O-N, and the latter one is attributed to surface adsorbed (NO_x_, NH_3_) and/or nitrogen trapped in the surface layers as *γ*-N_2_ [38,54,55,56]. Considering that no peaks that correspond to W_2_N or WN were observed in the XRD patterns, we confirmed the substitution of O in WO_3_ by N element and the formation of W-O-N banding. In the high-resolution XPS spectra, the S 2p (Figure 3D) peak at 168.7 eV was observed for WO_3_−15 (no signals for the two other samples), and it is assigned to the S 2p orbits in the +6 oxidation state [40,57]. The formation of W-S bonding instead of W-O bonding can be confirmed by the following two reasons: (1) the binding energy of 168.7 eV for W-S is different from that of 169.9 eV for the SO_4_^2−^, (2) S^2−^ doping may only occur with difficulty because the S^2−^ radius (1.70 Å) is significantly larger than O^2−^ (1.22 Å). Generally, the larger the ionic radius is, the doping would be more difficult to occur due to higher formation energy. Therefore, the replacement of W^6+^ by S^6+^ is more favorable than replacing O^2−^ with S^2−^. Furthermore, the XPS results also demonstrate that the S-N-WO_3_ could be formed when the n_W_:n_(NH4)2S_ ratio was over 1:5. Compared to that of the WO_3_−0, the positive shifts of 0.4 eV and 0.8 eV for W 4f and O 1s can be seen, which is attributed to the electron transfer from the dopant energy level to the CB of WO_3_. It is considered that this transfer can be benefitial to improving the optical properties of WO_3_.

**Figure 4:**
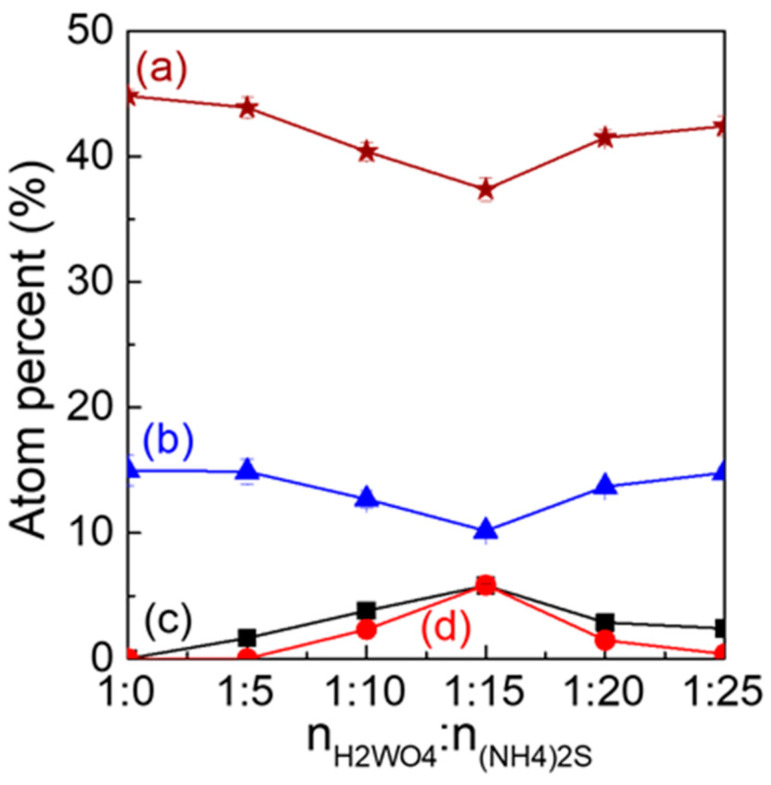
Plots of the contents of (a) O, (b) W, (c) N, and (d) S versus the addition of (NH_4_)_2_S.

To further reveal the mechanism of S and N co-doped WO_3_, it is necessary to discuss the influence of the n_W_:n_(NH4)2S_ ratio on the content of each element into the WO_3_ lattice (Figure 4). The contents of (a) O, (b) W, (c) N, and (d) S were calculated from XPS data (Figure 3 and Table 1). For WO_3_−0, the atom percent of W and O were 14.99% ± 1.2 and 44.82% ± 0.3, respectively. For WO_3_−5, no S element was doped into the WO_3_ lattice, only N element (1.64% ± 0.15). Compared to WO_3_−0, almost no change was observed for the W content (14.97% ± 1.0), but a decreasing trend was seen for the O (43.89% ± 0.8) content. As increasing the ratios from 1:5 to 1:15, the N content increased from 1.64% ± 0.15 to 5.82% ± 0.12, but the O content decreased from 40.4% ± 0.7 to 37.37 ± 0.8. It suggests that the higher n_W_:n_(NH4)2S_ ratios could lead to more oxygen defects due to N doping. Special attention should be paid to the change trend of W content, which decreased with the appearance of the S element from 1:10 (12.7% ± 0.7) due to the substitution of W^6+^ by S^6+^. The significantly higher contents for both N (5.82% ± 0.12) and S (5.85% ± 0.18) were obtained at 1:15 than at other ratios. Such high N and S contents can improve the absorption of visible light to further narrow the bandgap of WO_3_. Thereafter, the increase of atom percent for W and O and decrease for S and N was observed at higher n_W_:n_(NH4)2S_ ratios, and it may correspond to limitations in the substitution capacity of the WO_3_ lattice.

### 3.2. The Optical Properties of S-N-WO_3_

**Figure 5:**
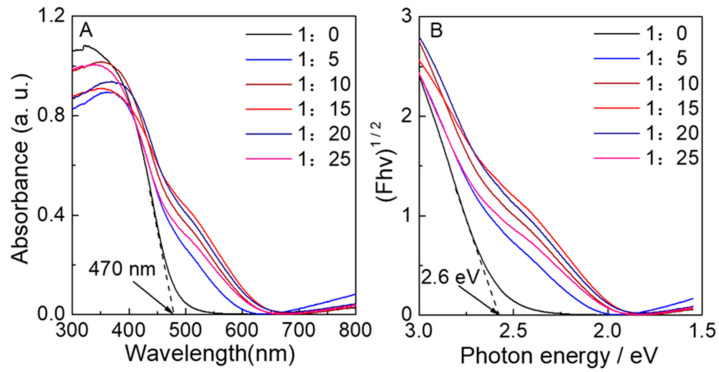
(**A**) UV-Visible DRS and (**B**) Tauc plots based on UV-Visible DRS of (black) WO_3_−0, (blue) WO_3_−5, (wine) WO_3_−10, (red) WO_3_−15, (navy) WO_3_−20, and (pink) WO_3_−25.

The DRS and the corresponding Tauc plots for the WO_3_ samples with changes in the ratio of n_W_:n_(NH4)2S_ are exhibited in Figure 5. As shown in Figure 5A, the WO_3_-0 can only absorb light below 470 nm. However, a significant redshift in the absorption edge with new shoulders appearing at 470–650 nm can be seen in N-doped WO_3_ or the S-N co-doped one. It was found that the absorption properties increased when increasing the ratio of n_W_:n_(NH4)2S_ below 1:15, and then they decreased when further increasing the addition of (NH_4_)_2_S. Absorption above 700 nm was observed for S-N-WO_3_ samples due to the formation of lattice defects caused by doping, in contrast to the negligible absorption for neat WO_3_. Furthermore, Tauc plots based on DRS data are shown in Figure 5B. The bandgap was determined by this technique in different materials [58,59,60]. It was reported that WO_3_ has an indirect optical bandgap. The Tauc plots for WO_3_−0 provided the absorption energy of 2.64 eV, which is in agreement with the bandgap energy of WO_3_ reported previously [11]. The Tauc plots for S-N-WO_3_ samples exhibited two different slopes due to the appearance of the new shoulders. Therefore, the estimated band energies for S-N-WO_3_ samples were obtained from the slopes, as displayed in Table 1. For WO_3_−5, the bandgap was reduced because a new intermediate N 2p orbital could be formed between the CB and the VB owing to N doping. It was observed that, in WO_3_ co-doped with S and N, the bandgap further decreased due to the formation of an intrabandgap above the VB edge and a dopant energy level below the CB of WO_3_.

**Figure 6:**
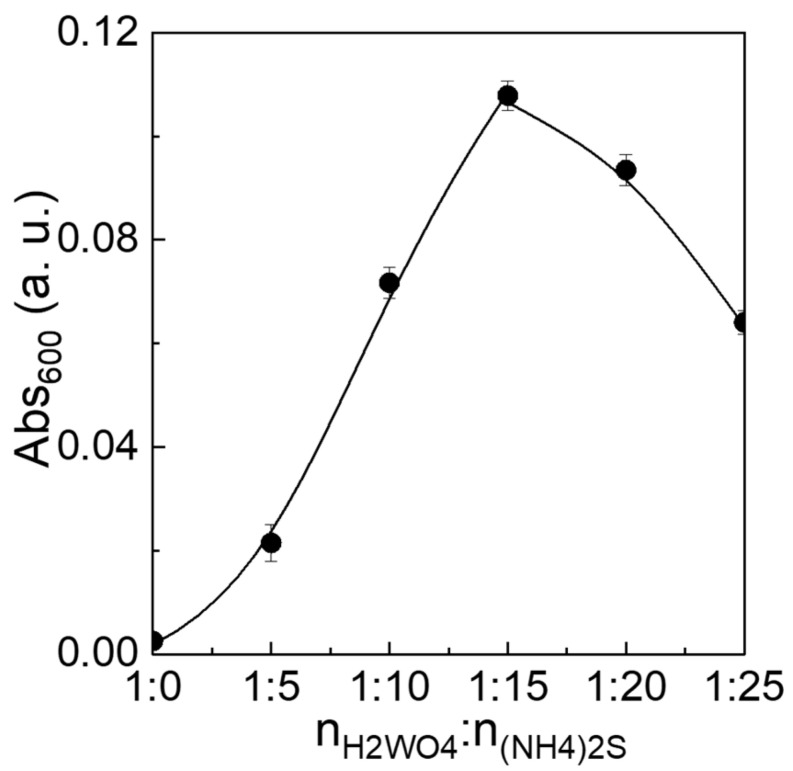
The absorbance values at 600 nm for the synthesized materials (WO_3_−0, WO_3_−5, WO_3_−10, WO_3_−15, WO_3_−20, and WO_3_−25).

Figure 6 is the relation between the absorbance value at 600 nm (Abs_600_). The Abs_600_ value is a measure of the increase/decrease of the shoulders at 470–650 nm. Compared with WO_3_−0, the Abs_600_ increased from 0.02 to 0.11 with an increase in the ratio of n_W_:n_(NH4)2S_ from 1:5 to 1:15, and, thereafter, decreased over 1:15 to 0.06 at 1:25. The dependency of Abs_600_ on the n_W_:n_(NH4)2S_ ratio agrees to the cases of the N and S content (Figure S1), indicating that the longer wavelength absorption due to the shoulders can be attributed to doping of N and S into a WO_3_ lattice.

### 3.3. Photoelectrocatalytic Properties

**Figure 7:**
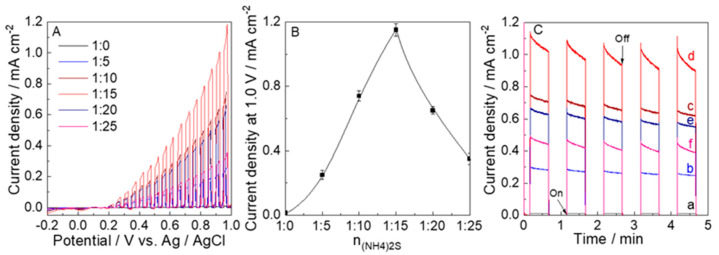
(**A**) Linear sweep voltammograms (LSV), (**B**) the plots of photocurrent density versus the addition of (NH_4_)_2_S, and (**C**) time course of the photocurrent at 0.68 V vs. Ag/AgCl (1.23 V vs. RHE) of the (a) WO_3_−0, (b) WO_3_−5, (c) WO_3_−10, (d) WO_3_−15, (e) WO_3_−20, and (f) WO_3_−25 electrodes with visible-light irradiation chopped in a 0.1 M phosphate buffer solution of pH 6.0 with visible-light irradiation (λ > 450 nm, 100 mW cm^−2^).

The LSVs for these electrodes calcined at 450 °C were measured with chopped visible light irradiation to study their PEC water oxidation performance. The photoanodic currents of these electrodes were observed above 0.1 V vs. Ag/AgCl due to water oxidation. The photocurrent of 1.15 mA cm^−2^ at 1.0 V for WO_3_−15 was the highest in comparison to other samples. Moreover, as shown in Figure 7B, the dependency of the photocurrent at 1.0 V on the n_W_:n_(NH4)2S_ ratio for each electrode is in agreement with the N and S contents. Figure 7C exhibits that the photocurrent at 0.68 V vs. Ag/AgCl (1.23 V vs. RHE) under visible-light irradiation chopped was stable during PEC water oxidation (5 min) for these electrodes. The photocurrent of the WO_3_−15 electrode (1.0 mA cm^−2^) was higher than those of the WO_3_−0, WO_3_−5, WO_3_−10, WO_3_−20, and WO_3_−25 by a factor of 83 (0.012 mA cm^−2^), 3.6 (0.28 mA cm^−2^), 1.4 (0.71 mA cm^−2^), 1.6 (0.62 mA cm^−2^), and 2.3 (0.44 mA cm^−2^), respectively.

**Figure 8:**
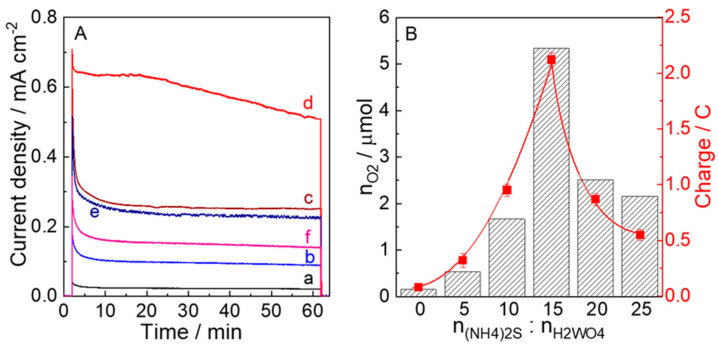
(**A**) Photocurrent density versus time profiles during PEC water oxidation in a 0.1 m phosphate buffer solution of pH 6.0 at 0.68 V vs. Ag/AgCl (1.23 V vs. RHE) and (**B**) O_2_ evolution amounts (n_O2_) and charge amounts during the 1 h photoelectrocatalysis upon visible-light irradiation (*λ* > 450 nm, 100 mWcm^−2^) using (a) WO_3_−0, (b) WO_3_−5, (c) WO_3_−10, (d) WO_3_−15, (e) WO_3_−20, and (f) WO_3_−25 electrodes.

Photoelectrocatalysis was conducted under the visible light irradiation (*λ* > 450 nm, 100 mW cm^−2^) at potentiostatic conditions of 0.5 V vs. Ag/AgCl (1.05 V vs. RHE) in a 0.1 M phosphate buffer (pH 6.0) for 1 h using electrodes calcined at 450 °C (Figure 8A). A higher photoanodic current due to water oxidation was observed for the WO_3_−15 electrode. Compared with the electrodes prepared at other n_W_:n_(NH4)2S_ ratios, the highest charge amount passed and the amount (n_O2_) of O_2_ evolved during the 1 h photoelectrocatalysis for WO_3_−15 were 2.12 C and 5.36 mmol (98% Faradaic efficiency), respectively (Figure 8B and Table S2). These results clearly prove that the doping of S and N enhances the PEC performance of WO_3_−15 in application to water oxidation.

**Figure 9:**
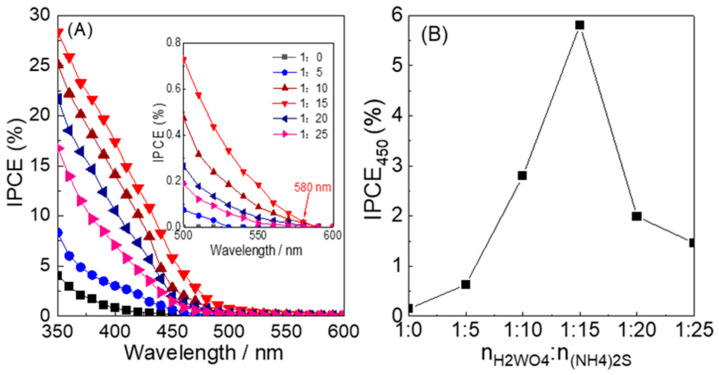
(**A**) Action spectra of IPCE of the (black) WO_3_−0, (blue) WO_3_−5, (wine) WO_3_−10, (red) WO_3_−15, (navy) WO_3_−20, and (pink) WO_3_−25 electrodes. (**B**) Plots of IPCE values at 450 nm versus the n_W_:n_(NH4)2S_ ratio for the synthesized materials (WO_3_−0, WO_3_−5, WO_3_−10, WO_3_−15, WO_3_−20, and WO_3_−25).

The action spectra of IPCE for these electrodes are shown in Figure 9. In Figure 9A, for WO_3_−0, the photocurrent was not observed above 470 nm, which is consistent with the bandgap energy of WO_3_. For the WO_3_−5 electrode, the onset wavelength for photocurrent generation was at least 520 nm, which, due to N doping, is significantly longer than that of WO_3_−0. The energy of the onset wavelength for WO_3_−5 (520 nm, 2.38 eV) was lower than the main bandgap excitation for WO_3_−5 (2.43 eV). This suggests that the photocurrent was generated based on the bandgap excitation, and the bandgap excitation occurs through collateral excitation from intermediate N 2p orbital to CB for the WO_3_−5 electrode. The onset wavelengths for WO_3_−10, WO_3_−15, WO_3_−20, and WO_3_−25, due to the S and N co-doping, are considerably shifted to the wavelengths (580 nm) longer than that of single N-doped WO_3_−5. However, for all of S-N-WO_3_ electrodes, the photocurrent at longer wavelengths longer than 580 nm could not be detected due to the limited current detection level of the employed apparatus. For the electrodes prepared at different n_W_:n_(NH4)2S_ ratios, the IPCE values at 450 nm (IPCE_450_) are shown in Figure 9B; the IPCE_450_ for WO_3_−5 electrode (0.63%) was 4.2 times higher than that of WO_3_−0 (0.15%), basically due to the formation of the formation of N doping_._ It precipitously increased at the ratios of 1:5 to 1:15, indicating that S and N co-doping plays a positive role in not only the increase in the onset wavelength but also in the increase in the IPCE_450_. The maximum IPCE_450_ of WO_3_−15 (5.81%) was obtained, which was 9.2 times higher compared to that of the WO_3_−5 electrode due to co-doping by S and N. It is suggested that the highest contents of S and N into WO_3_ lattice can effectively increase the electron transport rate and further inhibit recombination of electron-hole pairs in the film. When increasing the n_W_:n_(NH4)2S_ ratios, the IPCE_450_ for WO_3_−20 and WO_3_−25 reduced to 1.99% and 1.46%, respectively. However, they were still higher than that of the WO_3_−5 electrode. The relationship between IPCE_450_ and n_W_:n_(NH4)2S_ ratio is consistent with the Abs_600_ value in DRS data (Figure 6), indicating that the S and N co-doping is responsible for the lengthening of the onset wavelength for PEC water oxidation.

## 4. Conclusions

Nitrogen and sulfur co-doped crystalline WO_3_ was synthesized by thermal decomposition of (NH_4_)_2_S-derived precursor, in which (NH_4_)_2_S acted as a sulfur source, as well as the nitrogen source for doping. The addition of (NH_4_)_2_S has an effect on the physiochemical properties, and the performance of PEC water oxidation of the WO_3_-0 and S-N-WO_3_ electrodes was investigated to characterize the co-doping of S and N into the WO_3_ lattice and reveal the mechanism of superior performance for PEC water oxidation using the S-N-WO_3_ photoanode. S-N-WO_3_ exhibited the optimum n_W_:n_(NH4)2S_ ratio at 1:15 for the high concentration of both S and N elements. The S and N co-doping is responsible for the significant redshift in the absorption edge, with a new shoulder appearing at 470–650 nm compared to that of WO_3_−0. The S-N-WO_3_ photoanode is able to utilize visible light at wavelengths below 580 nm for PEC water oxidation, in contrast to the WO_3_−0 photoanode being able to work below 470 nm. The IPCE (5.81%) at 450 nm for S-N-WO_3_ photoanode calcined at 450 °C was higher than that (0.15%) for WO_3_−0 by 38.7 times due to the co-doping of S and N. The S-N-WO_3_ photoanode is expected to be applied for PEC water splitting cell as an artificial photocatalyst to improve the solar energy conversion efficiency.

## Figures and Tables

**Table 1:** Summary of physicochemical properties of various WO_3_ samples.

Samples	n_H2WO4_:n_(NH4)2S_	Molar Ratio of n_N_:n_W_ ^(^^a)^	Molar Ratio of n_S_:n_W_ ^(b^^)^	Absorption Energies ^(c)^ (eV)
WO_3_−0	1:0	1:0	1:0	2.64, -
WO_3_−5	1:5	0.19:1	1:0	2.44, 2.10
WO_3_−10	1:10	0.57:1	0.05:1	2.37, 2.02
WO_3_−15	1:15	1.64:1	0.19:1	2.16, 1.95
WO_3_−20	1:20	0.31:1	0.07:1	2.34, 1.97
WO_3_−25	1:25	0.28:1	0.04:1	2.39, 1.98

^(a)^,^(b)^ The local content of N and S contents were provided from EDS measurement. ^(c)^ The transition energies were given by Tauc plots of the samples based on DRS measurement.

## Supplementary Materials

The following supporting information can be downloaded at: https://www.mdpi.com/article/10.3390/nano12122079/s1, Figure S1: (A) Relationship between the relative contents of N, S and n_W_:n_(NH4)2S_ ratio; Figure S2: (A) the XPS survey spectrum and (B) XPS spectra in (A) W 4f, (B) O 2p regions for WO_3_−0; Table S1: Atomic percent of surface W, O, N, and S estimated by XPS; Table S2: Summary of PEC water oxidation in a 0.1 M phosphate buffer solution (pH 6.0) for 1 h using different WO_3_ electrodes calcined at 450 °C.
