# Supplementary material for: Fabrication of an Efficient N, S Co-Doped WO3 Operated in Wide-Range of Visible-Light for Photoelectrochemical Water Oxidation
Source: Nanomaterials (Basel). 2022 Jun 16;12(12):2079. doi: 10.3390/nano12122079 (PMC9228223; doi:10.3390/nano12122079)
Supplement: Supplementary file 1 [file nanomaterials-12-02079-s001.zip › nanomaterials-1753574-supplementary.pdf]

Supplementary Materials

Fabrication of an Efficient N, S Co-Doped WO_3 Operated in Wide-Range of Visible-Light for Photoelectrochemical Water Oxidation

Dong Li [1,*], Fachao Wu [2], Caiyun Gao [2,*], Hongfang Shen [1], Fei Han [1], Fenglan Han [1,3] and Zhanlin Chen [1]

[1] School of Material Science and Engineering, North Minzu University, Yinchuan 750021, China; shen_hongfang@nun.edu.cn (H.S.); hanfei@nun.edu.cn (F.H.); 2002074@nun.edu.cn (F.H.); 18995091369@163.com (Z.C.)
[2] Chemical Science and Engineering College, North Minzu University, Yinchuan, 750021, China; wufachao1997@163.com
[3] International Scientific & Technological Cooperation Base of Industrial Waste Recycling and Advanced Materials, Yinchuan 750021, China
* Correspondence: lidong@nun.edu.cn (D.L.); caiyun-gao@nun.edu.cn (C.G.)

Table S1. Atomic percent of surface W, O, N and S estimated by XPS.

Samples	W (%)	O (%)	N (%)	S (%)
WO_3–0	14.99±1.2	44.82 ± 0.3	0	0
WO_3–5	14.97±1.0	43.89 ± 0.8	1.64 ± 0.15	0
WO_3–10	12.7 ± 0.7	40.4 ± 0.7	3.81 ± 0.16	2.32 ± 0.13
WO_3–15	10.17 ± 0.2	37.37 ± 0.9	5.82 ± 0.12	5.85 ± 0.18
WO_3–20	13.7 ± 0.2	41.49 ± 0.6	2.88 ± 0.15	2.46 ± 0.14
WO_3–25	14.81 ± 0.5	42.41 ± 0.8	2.4 ± 0.2	0.36 ± 0.13

Table S2. Summary of PEC water oxidation in a 0.1 M phosphate buffer solution (pH 6.0) for 1 h using different WO_3 electrodes calcined at 450 °C.

Samples	Charge /C	n_{O2} /μmol	F.E.$_{O2}$ [(a)] (%)	n_{H2} [(b)] /μmol	F.E.$_{H2}$ [(c)] (%)
WO_3–0	0.08	0.11	54	0.34	83
WO_3–5	0.32	0.83	91	1.77	91
WO_3–10	0.95	2.37	97	4.89	99
WO_3–15	2.12	5.36	98	11.0	100
WO_3–20	0.87	2.06	92	4.38	98
WO_3–25	0.55	1.35	92	2.82	97

[(a)] Faradic efficiency of O_2 evolution. [(b)] n_{H2} is the amount of H_2 evolved in the Pt counter electrode compartment. [(c)] Faradic efficiency of H_2 evolution.

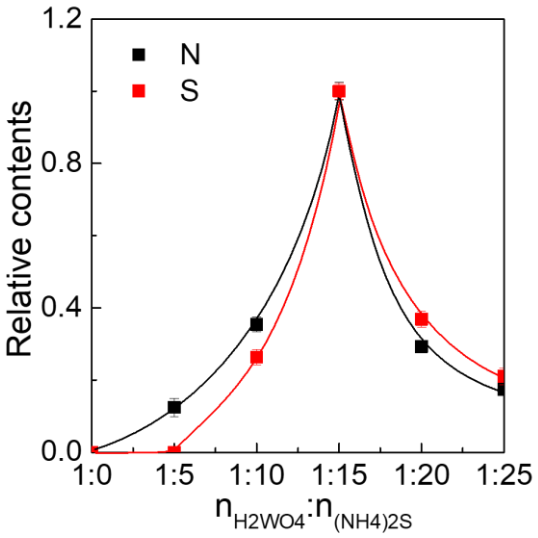

Figure S1. Relationship between the relative contents of N, S and n_W:$n_{(NH4)2S}$ ratio. The relative N and S contents were measured in EDS data (Table 1) and normalized by the highest contents for n_W:$n_{(NH4)2S}$ ratio of 1:15.

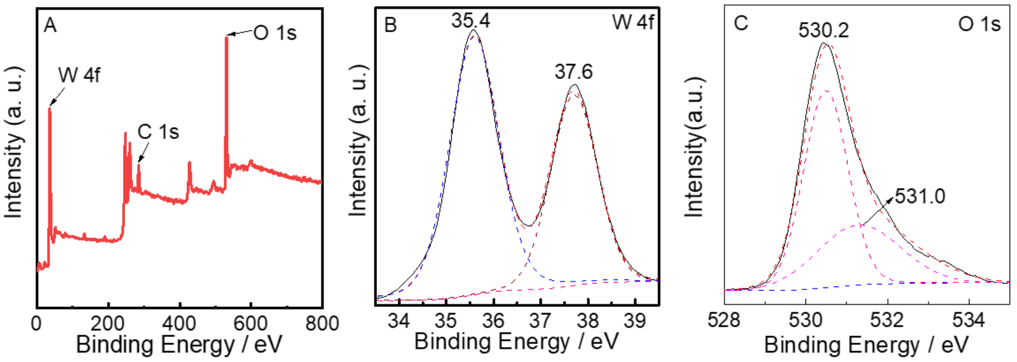

Figure S2. (**A**) the XPS survey spectrum and XPS spectra in (**B**) W 4f, (**C**) O 2p regions for WO_3–0. The XPS measurements were calibrated in reference to C 1 s peak fixed at 284.2 eV.
